# Toll-Like Receptor 9 Alternatively Spliced Isoform Negatively Regulates TLR9 Signaling in Teleost Fish

**DOI:** 10.1371/journal.pone.0126388

**Published:** 2015-05-08

**Authors:** Frank Fang-Yao Lee, Hsiang-Chieh Chuang, Nai-Yu Chen, Govindarajulu Nagarajan, Pinwen Peter Chiou

**Affiliations:** 1 Marine Research Station, Institute of Cellular and Organismic Biology, Academia Sinica, Taipei, Taiwan; 2 Molecular and Biological Agricultural Sciences, Taiwan International Graduate Program, Academia Sinica, Taipei, Taiwan; 3 Department of Life Science, National Taiwan University, Taipei, Taiwan; 4 Biotechnology Center, National Chung-Hsing University, Taichung, Taiwan; National Cheng Kung University, TAIWAN

## Abstract

Toll-like receptor 9 (TLR9) recognizes and binds unmethylated CpG motifs in DNA, which are found in the genomes of bacteria and DNA viruses. In fish, *Tlr9* is highly diverse, with the number of introns ranging from 0 to 4. A fish *Tlr9* gene containing two introns has been reported to express two alternatively spliced isoforms, namely gTLR9A (full-length) and gTLR9B (with a truncated Cʹ-terminal signal transducing domain), whose regulation and function remain unclear. Here, we report a unique regulatory mechanism of gTLR9 signaling in orange-spotted grouper (*Epinephelus coioides*), whose *gTlr9* sequence also contains two introns. We demonstrated that the grouper *gTlr9* gene indeed has the capacity to produce two gTLR9 isoforms via alternative RNA splicing. We found that gTLR9B could function as a negative regulator to suppress gTLR9 signaling as demonstrated by the suppression of downstream gene expression. Following stimulation with CpG oligodeoxynucleotide (ODN), gTLR9A and gTLR9B were observed to translocate into endosomes and co-localize with ODN and the adaptor protein gMyD88. Both gTLR9A and gTLR9B could interact with gMyD88; however, gTLR9B could not interact with downstream IRAK4 and TRAF6. Further analysis of the expression profile of g*Tlr9A* and g*Tlr9B* upon immune-stimulation revealed that the two isoforms were differentially regulated in a time-dependent manner. Overall, these data suggest that fish TLR9B functions as a negative regulator, and that its temporal expression is mediated by alternative RNA splicing. This has not been observed in mammalian TLR9s and might have been acquired relatively recently in the evolution of fish.

## Introduction

Pathogen recognition is a critical step for the host to mount a successful immune response. Such recognition can be facilitated by pattern recognition receptors (PRRs) that sense danger signals or non-self molecules. PRRs either interact with these molecules in intracellular compartments where they should not be normally present (e.g., self-DNA within the endosome) or by recognizing pathogen-associated molecular patterns (PAMPs) that are found exclusively (e.g., lipopolysaccharides on the surface of gram-negative bacteria) or mostly (e.g., unmethylated CpG DNA motif) in pathogens.

Toll-like receptors (TLRs) were the first class of PRRs to be identified, and the main mechanisms of TLR signaling pathways are well conserved from the simplest multicellular organism *Caenorhabditis elegans* to mammals [1]. Aligned from the N’ terminus to the C’ terminus, the TLR molecule contains the following domains: a leucine-rich repeat (LRR) domain for interacting with specific PAMP(s), an intermediate transmembrane region for membrane localization, and a Toll-interleukin I receptor-resistance (TIR) domain to transduce downstream signals [2]. Structural analysis of the human TLR10 TIR domain has shown that it contains five β-sheets (βA–βE) and five α-helices (αA–αE). These secondary structures are connected by loops (AA–EE). When TLR10 binds to a PAMP, it dimerizes via a TIR-TIR interaction through surfaces formed by BB loops, DD loops, and the αC helix [3]. PAMP binding to TLRs triggers downstream signaling via the recruitment of adaptor proteins to their TIR domains. In mammals, four adapter proteins contain a TIR domain and are associated with the initiation of TLR signaling. These include myeloid differentiation primary response 88 (MyD88), TIRAP/Mal, TRIF/TICAM1, and TRAM/TICAM2. Of these, MyD88 is utilized by all TLRs except for TLR3, which recruits TRIF as an adaptor protein. These adaptor proteins either transmit signals from TLRs to downstream effectors, or serve as co-adaptors that bridge the interaction between the receptor and adaptor proteins [4].

TLR9 is an intracellular receptor that recognizes unmethylated CpG DNA originating from the genomes of bacteria, DNA viruses, or from self-DNA generated under pathological conditions [5]. In unstimulated cells, TLR9 is mainly found within the endoplasmic reticulum (ER). Upon uptake of agonist, TLR9 translocates from the ER into the endosomal compartment, where it can subsequently bind to its agonist. This interaction triggers the recruitment of the adaptor protein MyD88, which contains an N-terminal death domain and a C-terminal TIR domain. Once recruited by TLR9, MyD88 forms a large protein complex, referred to as the “Myddosome,” which is required for downstream signaling [6–8]. This series of events ultimately results in the activation of the transcription factors IFN-regulatory factor 7 (IRF7) and NF-κB, which induce the expression of type I interferons (IFNs) and inflammatory cytokines such as IL-1β, especially. IFN can further stimulate the expression of interferon-stimulated genes (ISGs) such as Mx, which act to limit viral infection [2].

In vertebrates, PRR-regulated innate immunity is likely to be more important to fish than to mammals, as the adaptive immune system in fish is primitive compared to its counterpart in mammals [9]. It is, therefore, not surprising that fish have evolved more complexity in the repertoire and function of their TLRs. In general, fish TLRs are similar to their counterparts in mammals. Previous studies on the zebrafish (*Danio rerio*) and pufferfish (*Fugu rubripes*) genomes revealed that fish share major PRRs and signaling pathways with mammals, although some differences exist [10–12]. For example, TLR4 is the only TLR in mammals that is known to recognize bacterial lipopolysaccharides (LPS); however, there is no evidence of a functional TLR4 in fish [9,13–17]. The lack of a functional TLR4 indicates that fish might be equipped with alternative LPS-sensing machinery. However, some species of fish possess an expanded repertoire of TLRs that have only been identified in mammals, such as *tlr19* and *tlr20* in zebrafish, and *Tlr5S* and *Tlr23* in pufferfish [9]. Different TLR isoforms are also found in abundance in fish. For example, the lack of genes encoding the major histocompatibility complex (*MHC*) class II, *CD4*, and invariant chain (*Ii*) in Atlantic cod (*Gadus morhua*), is compensated for by the existence of an expanded repertoire of MHC class I and *Tlr* genes [18]. Discrepancy has also been reported at genetic and functional levels in Tlr3 signaling in zebrafish, in which the downstream activation of NF-κB is not dependent on the interaction between the adaptor protein, Trif, and Tnfr-associated factor 6 (Traf6), as occurs in mammalian TLR3 signaling. Sequence analysis has revealed that zebrafish Trif does not contain a Traf6-binding site, which is well conserved in mammalian TRIF genes [19].

**Fig 1:**
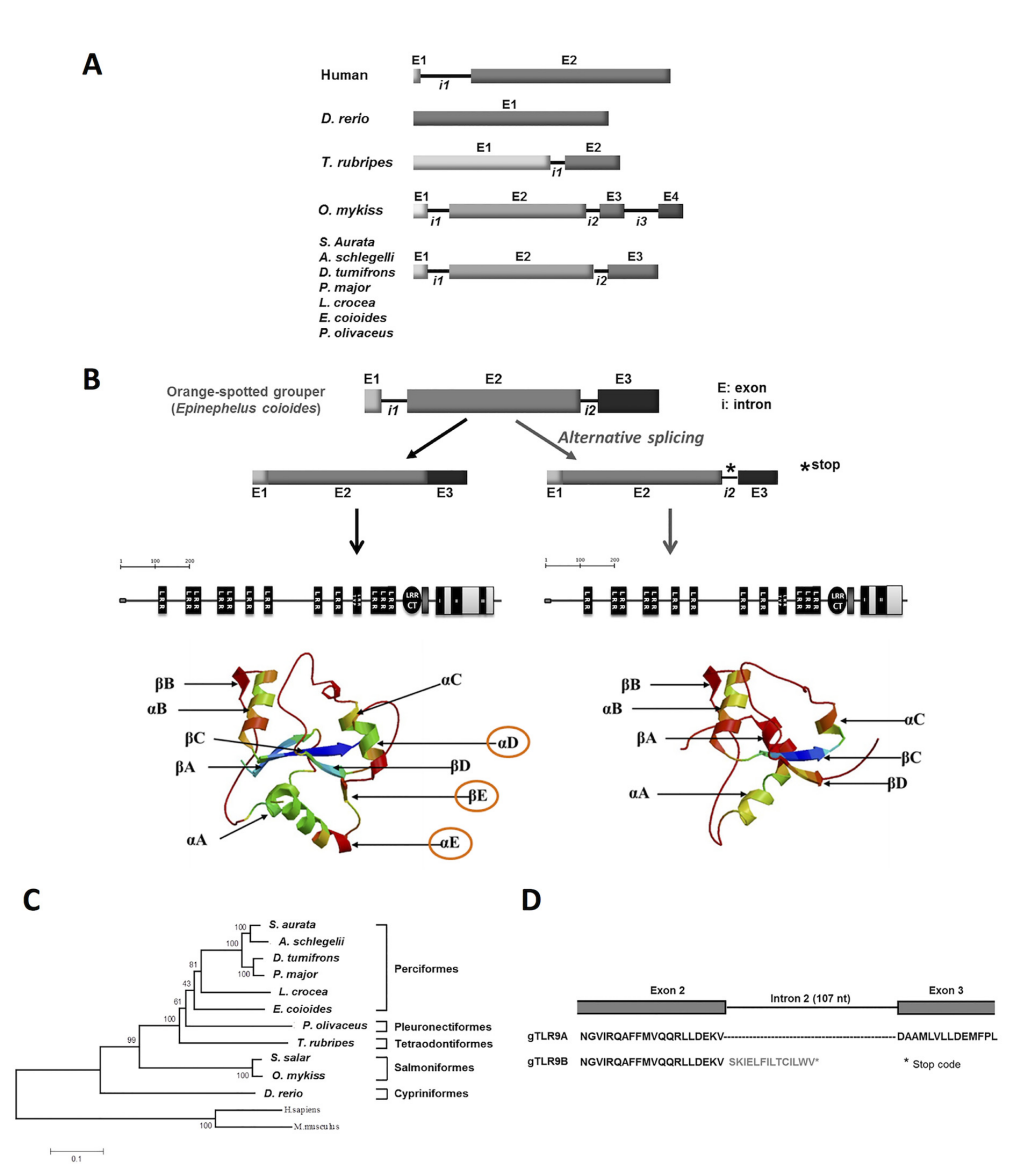
Isoforms of grouper *Tlr9 (gTlr9)*. *A*, Structures of fish *Tlr9* genes. There are four known structures of the *Tlr9* gene in teleosts. The grouper *Tlr9* gene contains three exons and two introns. To date, the 2-intron *Tlr9* gene has only been found in members of the orders *Perciformes* and *Pleuronectiformes*. *B*, Two mRNA species derived from the grouper *Tlr9* gene. Grouper *Tlr9* pre-mRNA can be spliced into two isoforms, namely *gTlr9A* and *gTlr9B*. The nucleotide sequence of *gTlr9B* contains an insertion at intron 2, resulting in a translational product with a truncated TIR domain lacking the conserved Box III. *C*, Phylogenetic relationship of the *Tlr9* gene sequence from grouper and other fish species. Grouper TLR9A is most closely related to its counterparts in the orders *Perciformes* and *Pleuronectiformes*. *D*, Differences in the amino acid sequence between gTLR9A and gTLRB. The amino acid sequence of gTLR9A is encoded by an open reading frame constituted by exons 1, 2, and 3. The alternative isoform gTLR9B contains amino acids that are encoded by exons 1 and 2, followed by 14 amino acids that are encoded by intron 2. LRR: leucine-rich repeat; LRR CT: LRR at the C′-terminus.

Differences in the *Tlr9* family between fish and mammals can be observed in the genome organization and in transcriptional products. *Tlr9* in teleost fish has been cloned and characterized in several species, including common carp, flounder, sea bream, rainbow trout and yellow croaker [9,20–26]. In mammals, a single intron in the translational start codon is present in the *TLR9* genes [27]. In teleosts, the number of introns has been reported to range from zero to four (Fig 1A) [11,12,21]. Fish *Tlr9* genes with two-intron organization have been reported to undergo intron-retention alternative splicing (AS) in intron 2, resulting in a C-terminal truncated TLR9B in addition to the full-length TLR9A [21,24]. Widespread AS events have been reported in the expression of innate immune genes in mouse TLR signaling cascades, as exemplified by the alternatively spliced variants of *TLR4*, *TICAM1*, *TOLLIP*, *RAC1*, *IRAK1*, *2*, and *4*, *MAPK14/p38*, *ATF2*, and *STAT1*. Functional study of splice variants in mouse macrophages in response to inflammatory mediators revealed a common role for variant proteins in the control of inflammatory signaling [28]. Thus, it is reasonable to speculate that alternatively spliced TLR9B in fish may play a regulatory role in TLR9 signaling. However, this has yet to be verified.

In the current study, we examined AS-mediated TLR9 signaling in grouper (*Epinephelus* spp.), a coral marine fish with important economic value. We show that the grouper *Tlr9* gene also possesses two introns and can produce two alternatively spliced mRNA and protein isoforms, namely gTLR9A (full-length TLR9) and gTLR9B (containing a truncated TIR domain). We also examined the expression, ligand interaction, downstream recruitment of signaling complexes, and function of gTLR9B in grouper cells in response to stimulation with TLR9 agonists. These analyses reveal a unique control mechanism in fish TLR9 signaling, in which the truncated gTLR9B protein functions as a negative regulator, whose temporal expression is mediated at the mRNA level by AS.

## Materials and Methods

### Cells and Animals

Grouper kidney cells (GK cells) were grown in Leibovitz’s L-15 media supplemented with 10% fetal bovine serum at 28°C [29]. Human embryonic kidney HEK293T (ATCC cat. CRL-3216) cells were cultured in Dulbecco’s modified Eagle medium supplemented with 10% fetal bovine serum in a humidified CO_2_ incubator at 37°C. Orange-spotted grouper (*Epinephelus coioides*) were obtained from the National Cheng Kung University and were housed in a 40-ton holding tank at the density of 30 fishes per tank with constant aeration. The fresh seawater was changed, and the fish were fed daily. To analyze the tissue distribution of g*Tlr9* isoforms, tissue samples were collected from three fish euthanized with tricaine methanesulfonate (MS222; Sigma). The animal handling and experimental procedure was approved by the Institutional Animal Care and Use Committee (IACUC) of the Academia Sinica under the approved protocol# RFiZOOCP2008094.

### TLR9 agonists

Three agonists were used in this study: formalin-inactivated *Vibrio vulnificus*, *Escherichia coli* chromosomal DNA, and a synthetic CpG ODN. *V*. *vulnificus* were inactivated in 0.5% formalin (in PBS) with continuous shaking at room temperature for 24 h, followed by washing and resuspension in L15 medium at a final concentration equivalent to 3 OD_600_. The complete inactivation of *V*. *vulnificus* was verified by plating the suspension on LB plates. To generate bacterial DNA, purified chromosomal DNA from *E*. *coli* strain B (Sigma) was resuspended in L15 medium and sheared by passing through a 25-gauge needle six times to fragment the DNA into fine pieces. Transfection of *E*. *coli* DNA into GK cells was carried out with PolyJet (SignaGen Laboratories) by following the manufacturer’s protocol. A custom-designed type A CpG ODN was manufactured and purified by high-performance liquid chromatography, HPLC (Sigma).

### Cloning and sequence analysis of grouper *Tlr9* and *MyD88*

A PCR cloning strategy was initially adopted to clone partial fragments of the grouper *Tlr9* and *MyD88* by using degenerate primers that were based on conserved sequences obtained from the nucleotide sequence database at NCBI (http://www.ncbi.nlm.nih.gov/). The PCR-amplified products were subcloned into a T/A cloning vector, pCR3.1 (Invitrogen, USA), and sequenced. Primers that were specific to the new partial sequences were designed and used to obtain the 5′ and 3′ ends of the genes by using SMART RACE cDNA Amplification Kit (Clontech, USA), according to the manufacturer’s instructions. Using this approach, the full-length cDNAs of two grouper *Tlr9* mRNAs (namely *gTlr9A* and *gTlr9B*) and *gMyD88* were obtained. Primers specific to the 5′ and 3′ termini of *gTlr9A* and *gTlr9B* were generated to obtain the sequence of the *gTlr9* gene by PCR cloning. The primers used for cloning are listed in S1 Table. For the analysis of protein structure, known protein domains were analyzed by the SMART and SignalP3.0 programs.

### Plasmid constructs

Plasmids were constructed to assay the transcriptional and translational capacity of the grouper *Tlr9* gene. A quick-change PCR strategy was adopted to insert the grouper genomic *Tlr9* sequence into a eukaryotic expression vector, pcDNA3.1, to generate the plasmid pgTLR9-genomic. The same strategy was used to introduce 3× Flag tag into exon 3, or 4× HA tag into exon 2 in pgTLR9-genomic. These constructs were termed pgTLR9A-FLAG-genomic and pgTLR9B-HA-genomic, respectively. Plasmids encoding HA-tagged *gMyD88* cDNA and Flag-tagged *gTlr9A*, *gTlr9B* cDNA for subsequent co-immunoprecipitation were constructed by inserting *gMyD88* and g*Tlr9A/gTlr9B* cDNA into BamH1/XhoI sites of pcDNA3.1 NF2-HA, and BamH1 site of pcDNA3-3Flag vectors to generate pcDNA MyD88-HA, pcDNA3.1-9A, and pcDNA3.1-9B, respectively. The plasmid pEYFP-MyD88 was constructed by inserting *MyD88* cDNA into Sal1 and Bam HI sites of the vector pEYFP-C1 (Clontech). The identity of all plasmids was verified by DNA-sequencing.

### Transcription and translation of grouper *Tlr9* gene

To assay the transcriptional capacity of the grouper *Tlr9* gene, pgTLR9-genomic encoding the full-length grouper *Tlr9* gene sequence was transfected into HEK293T cells. A total of 5 × 10^5^ cells/per well were seeded onto 6-well plates one day prior to transfection. The transfection was performed with Lipofectamine 2000 (Invitrogen), following the manufacturer’s instructions. Six hours after transfection, the transfection medium was replaced with fresh serum-free Dulbecco’s modified Eagle medium, and cells were incubated for an additional 18 h before RNA extraction was performed. The levels of *gTlr9A* and *gTlr9B* mRNA were determined by RT-PCR with primers designed to flank alternatively spliced intron 2, as described below. To assess the transcriptional capacity of the grouper *Tlr9* gene, HEK293T cells were transfected with the pgTLR9A-Flag-genomic or pgTLR9B-HA-genomic plasmid encoding the gene sequence of grouper TLR9 tagged with Flag or HA for gTLR9A and gTLR9B, respectively. Twenty-four hours after transfection, total protein was harvested with RIPA buffer (Millipore, cat 20–188), and was subjected to separation by 8% SDS-PAGE, and transferred onto a PVDF membrane for western blotting.

### Expression profiling and quantitation of *gTlr9A* and *gTlr9B* transcripts

GK cells seeded onto a 6-well plate were pulse-treated with each individual agonist for 1 h. After pulse-treatment, the medium was removed, and the cells were washed in serum-free L15 three times prior to the addition of fresh L15 medium. At the indicated time points, the culture medium was harvested and the cells were frozen at -20°C until RNA extraction. The gene expression profiles of *gTlr9A* and *gTlr9B* were analyzed by RT-PCR, and the relative expression of each was determined by RT-quantitative PCR (RT-qPCR). Briefly, total RNA from tissues or from GK cells was prepared using TRIzol reagent (Invitrogen) following the manufacturer’s instructions. To remove any contaminating DNA, all RNA samples were further treated with DNase I (Promega, USA) prior to the second round of purification with TRIzol. First strand cDNA was synthesized from each RNA sample (3 μg) using SuperScript III reverse transcriptase (Invitrogen, USA) with oligo dT primer, following the recommendations of the manufacturer. The PCR reaction was carried out with a forward (5′-GAAGACAGTGTTTGTGCTGTCCAGCGGT-3′) and reverse primer (5′-GAGGTTATCTGATGACAATGCCATTCTC-3′), which are complementary to sequences residing in exons 2 and 3, respectively, and thus could be used to amplify both *gTlr9A* (product size: 274 bp) and *gTlr9B* (product size: 381 bp). Primer sets specific to *gTlr9A* or *gTlr9B* were used in RT-qPCR analysis. For *gTlr9A*, the primers used were as follows: forward 5′-GTTTGTGCTGTCCAGCGGT-3′, and reverse 5′-GCATAGCTGCATCCACCTTC-3′. For *gTlr9B*, forward 5′-GCGACTTCTGGACGAGAAGGT-3′, and reverse 5′-AACATGGCTACAACAGGATATGAATC-3′ primers were used. The reaction was conducted using 5 μL of the 10X-diluted RT products in a total volume of 50 μL consisting of 1X standard Taq polymerase buffer (Promega, USA), 1.5 mM MgCl_2_, 350 nM gene-specific primers, 200 μM dNTPs, 1X SYBR Green I (BMA, USA), and 2 units of Taq polymerase (Promega, USA). The thermocycling conditions used for the reaction were as follows: 94°C for 3 min, followed by 50 cycles consisting of 94°C for 15 s, 59°C for 15 s, and 72°C for 20 s. The reaction was performed on each sample in triplicate on a Rotor-Gene Q machine (QIAGEN, USA). Relative expression of each gene of interest was normalized to the β-actin gene respectively.

### Regulatory effect of gTLR9B on the downstream genes in the TLR9 pathway

GK cells were transfected with plasmids encoding gTLR9A (pcDNA3.1-9A) or gTLR9B (pcDNA3.1-9B) cDNAs and the plasmid pRL-TK encoding Renilla luciferase, which is used as a transfection control for Lipofectamine 2000 (Invitrogen). Eighteen hours post-transfection, the media was removed and the cells were pulsed-treated with PolyJet embedded ODN. At 3, 6, 12, and 24 h after pulse treatment, the media and total RNAs were collected for subsequent ELISA and RT-qPCR analysis. Triplicate samples were collected for each time point and treatment. ELISA was performed using 100 μL culture medium collected from each experiment, and the antigen was detected with a rabbit anti-grouper IL-1β antibody prepared in-house. The results of the ELISA assay were determined using a Varioskan Flash spectral scanning multimode plate reader (ThermoScientific). The specificity of the IL-1β antibody was verified using recombinant IL-1β protein (S2 Fig). RNAs were isolated using TRIzol reagent as described previously, and the levels of *Il-1*β and *Mx* expression were analyzed by RT-qPCR. The primers used for RT-qPCR are listed in S1 Table. All values from ELISA or RT-qPCR were normalized to the expression of *Renilla* luciferase mRNA.

### Subcelluar localization of gTLR9A, gTLR9B, and gMyD88

GK cells were transfected with pgTLR9A-Flag-genomic or pgTLR9B-HA-genomic together with pEYFP-MyD88 in 12-well plates. At 24 h after transfection, cultured cells were incubated with 20 nM of CpG ODN or Texas red-conjugated CpG ODN (Sigma) for 15 or 30 min. Stimulated cells were fixed with 4% formaldehyde at room temperature for 15 min, and were then permeabilized with 0.2% Triton X-100 in PBS. The blocking reaction was performed in PBS containing 5% bovine serum albumin (BSA) for 1 h at room temperature. Indirect immunofluorescence was performed with rabbit anti-FLAG antibody (Sigma, cat. no F7425), rabbit anti-HA antibody (Viogene, cat. no. AH1001), and goat anti-rabbit IgG conjugated to Texas Red (Invitrogen, cat. no T2767) diluted in PBS containing 5% BSA. Cell nuclei were counter stained with 4′,6-diamidino-2-phenylindole (DAPI; Molecular Probe). To label the early endosome, human transferrin conjugated to Alexa Fluor 633 (10 μg/mL, Invitrogen) was added into the culture media along with the ODN. Cells were mounted onto glass slides with PBS containing 0.1 g/mL 1,4-diazabicyclo-[2,2,2]octane (DABCO), 50% glycerol, and 0.1% Na_3_N. Images were acquired on a TCS-SP5-AOBS (Leica, Germany) inverted confocal microscope with excitation lasers at 405, 488, 594, and 633 nm.

### Immunoprecipitation and western blotting

HEK293 cells seeded onto 6-well dishes were transfected with pcDNA3.1-9A or pcDNA3.1-9B encoding *gTlr9A-Flag* or *gTlr9B-Fla*g cDNAs along with gMyD88-HA, at 37°C in a CO_2_ incubator for 24 h. The cells were then washed with PBS and collected by scraping from culture flasks. Collected cells were washed twice with cold PBS and then incubated in 1 mL lysis buffer (50 mM Tris pH7.4, 250 mM NaCl, 5mM EDTA, 50mM NaF, 1mM Na_3_VO_4_, 1% Nonidet P40, and 0.02% Na_3_N) together with 1mM PMSF, and 100 μL protease inhibitor cocktail (Sigma, cat. no. P-2714) for 30 min on ice. Cell lysates were cleared by centrifugation at 13,000 rpm for 10 min at 4°C, aliquoted, and stored at -80°C. Immunoprecipitation was performed with Dynabeads Protein G (Invitrogen), following the manufacturer’s recommendations. Briefly, 50 μL (1.5 mg) of Dynabeads were coated with 5 μg monoclonal anti-FLAG (Sigma, Cat.no. F1804) or monoclonal anti-HA (Sigma, Cat.no. H9658) diluted in 200 μL PBS w/ 0.02% Tween 20 for 10 min at room temperature with gentle rotation. Antibody-coated Dynabeads were crosslinked with bis(sulfosuccinimidyl)suberate (BS3; Thermo Scientific) to reduce non-specific binding. The antibody-coated and crosslinked beads were then washed in 200 μL PBS with Tween 20, and mixed with 800 μL cell lysate. The bead-lysate mixture was incubated with gentle rotation overnight at 4°C to allow co-immunoprecipitation of the target antigen. Proteins were separated by SDS-PAGE and then transferred to PVDF membranes. Membranes were blocked with 5% nonfat milk overnight at room temperature. Detection of HA- or Flag-tagged gMyD88 or gTLR9A/B was carried out by probing the membranes with rabbit anti-Flag (Sigma, cat. no. F7425) and rabbit anti-HA (Viogene, cat. no. AH1001) antibodies. Proteins involved in the formation of the TLR9 signaling complex were detected with rabbit anti-IRAK4 (LifeSpan, cat. no. LS-C164429) and rabbit anti-TRAF6 (Novus, cat. no. NB600-976). Protein bands were detected by incubating the probed membranes with HRP-conjugated goat anti-rabbit IgG (Sigma) and Immobilon western chemiluminescent horseradish peroxidase (HRP) substrate (Millipore). The chemiluminescent images were captured with a UVP BioSpectrum 500 Imaging System (UVP).

### RNA stability assay

To measure the relative stability of *gTlr9A* and *gTlr9B* mRNAs, GK cells were subjected to pulse stimulation with inactivated *V*. *vulnificus* for 1 h. Three hours post-stimulation, fresh serum-free L15 medium containing actinomycin D (Sigma) at a concentration of 1 μg/mL was added to the cells. Cells were incubated with actinomycin D for 0, 0.5, 1, 3, 9, and 12 h, the culture medium was removed, and the cells were frozen at -20°C until RNA extraction. The steady-state levels of *gTlr9A* and *gTlr9B* mRNA were measured by RT-qPCR as described above.

## Results

### Grouper *Tlr9* isoforms

Two isoforms of *gTlr9* cDNA, named *gTlr9A* and *gTlr9B*, were cloned from the head kidney of orange-spotted grouper. The full-length *gTlr9A* cDNA (NCBI ID: GQ358201.1) is 3800 nucleotides (nt) long, consisting of a 265-nt 5′-untranslated region (UTR), an open reading frame (ORF) encoding a protein of 1061 amino acids, and a 317-nt 3′-UTR (S1 Fig). The predicted protein domains in gTLR9A include an N-terminal domain containing a signal peptide (20 aa), a region of 14 leucine-rich repeats (LRRs), a transmembrane domain, and a C-terminal region with a typical TIR domain containing three conserved boxes (Box I, II, and III) that are important for downstream signaling (Fig 1B). Comparison of the amino sequence of gTLR9A with its counterparts in other vertebrates revealed moderate sequence identity with teleosts (from 51% identity with *Danio rerio* to 75% identity with *Dentex tumifrons*), and low sequence identity with mammals (from33% identity with *Felis catus* and *Mus musculus* to 34% with *Homo sapiens*, S2 Table). Phylogenetic analysis showed that grouper *Tlr9A* is closely related to its counterparts in the order *Perciformes*, which have *Tlr9* genes containing two introns (Fig 1C).

The full-length *gTlr9B* cDNA (NCBI ID: GQ358202.1) is 3907 nt long, and the composition is identical to that of *gTlr9A* except for an insertion of 107 nt near the 3′-terminus. To investigate the origin of the inserted sequence, we further cloned and sequenced the genomic sequence of *gTlr9*. Analysis of the grouper *gTlr9* gene sequence using the FSPLICE program (//www.softberry.com/) predicted that *gTlr9* contains three exons and two introns as follows: exon 1 (301 nt)—intron 1 (278 nt)—exon 2 (2978 nt)—intron 2 (107 nt)—exon 3(521 nt) (Fig 1B). The sequence of the 107-nt insertion in *gTlr9B* was identical to the sequence of intron 2, indicating that *gTlr9B* is a product of an intron-retention alternative splicing event that occurred during the processing of *gTlr9* pre-mRNA. The predicted translational product of *gTlr9B* mRNA varies from that of *gTlr9A* by the addition of 15 amino acids derived from intron 2, and a lack of amino acids encoded for by exon 3 (Fig 1D). Hence, gTLR9B contains all known structures and conserved motifs presented in the gTLR9A TIR domain except for the αD, βE, and αE structures of the connective DD loops, and the Box III situated within the αE helix (Fig 1B).

### Transcriptional and translational capacity of the *gTlr9* gene

**Fig 2:**
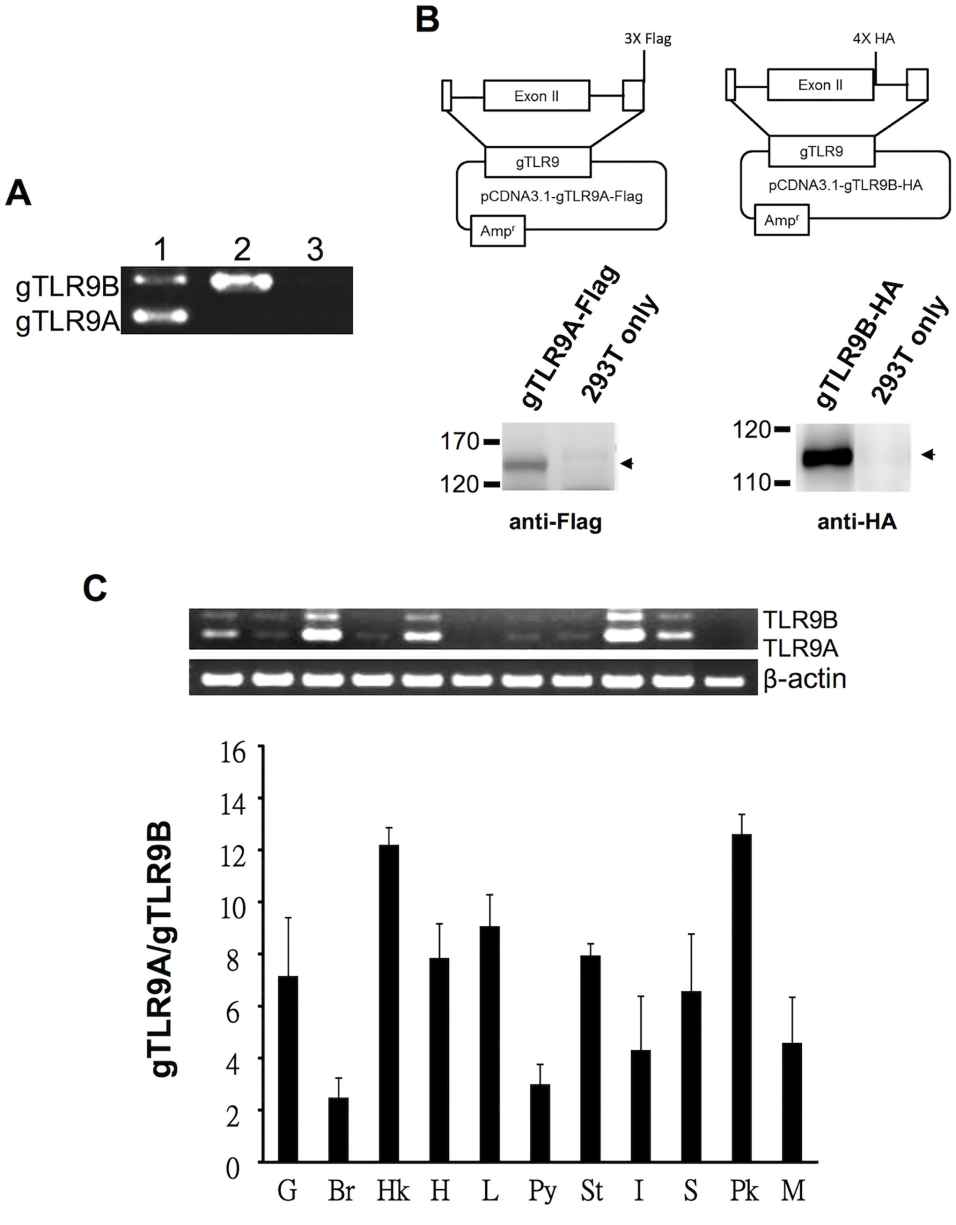
Transcriptional and translational capacity of the grouper *Tlr9* gene. *A*, Transcriptional activity of the *gTlr9* gene was assayed in HEK293T cells transfected with the plasmid pgTLR9-genomic, which contains the grouper *Tlr9* gene sequence. The expression of *gTlr9A* and *gTlr9B* mRNAs in transfected cells was detected by RT-PCR with primers flanking intron 2 of *gTlr9*. Lane 1, gTLR9-transfected HEK293T; lane 2, plasmid gTLR9; lane 3, non-transfected HEK293T. *B*, Translational activity of the grouper *Tlr9* gene was assayed in HEK293T cells transfected with a plasmid containing the *Tlr9* genomic sequence tagged with either Flag after exon 3 (gTLR9A-Flag) or HA in intron 2 (gTLR9B-HA). The expression of gTLR9A and gTLR9B proteins was confirmed by western blotting in cells transfected with gTLR9A-Flag and gTLR9B-HA, respectively. *C*, The tissue distribution and relative abundance of *gTlr9A* and *gTlr9B* transcripts were assayed by RT-PCR (upper panel) and RT-qPCR (lower panel) with three fish. The upper panel documents the data of one representative fish. In the lower panel, the RT-qPCR results are presented as the mean ± SD of the data from the three fish. Both mRNAs were predominately detected in immune-related tissues. St, stomach; Py, pyloric ceca; Sp, spleen; Pk, posterior (head) kidney; Br, brain; In, intestine; Hk, heard kidney; G, gills; L, liver; Hr, heart; M, muscle.

In teleost, TLR isoforms can be generated from a single gene by AS, or can be encoded by different genes as observed for trout soluble TLR5 [30]. To examine whether the grouper *Tlr9* gene is indeed capable of generating both transcriptional and translational products, we transfected HEK293T cells with a plasmid carrying the *gTlr9* genomic sequence (pgTLR9-genomic). We chose human HEK293T cell to test the transcription and translation capacity of *gTlr9* genome for better transfection efficiency as well as to avoid interference with the basal expression of *gTlr9A/9B* in GK cells (data not shown) As shown in Fig 2A, both *gTlr9A* and *gTlr9B* transcripts could be identified in the transfected cells by RT-PCR, demonstrating that the transcription of *gTlr9* can generate the AS product, *gTlr9B*. We constructed two further plasmids, pgTLR9A-Flag-genomic and pgTLR9B-HA-genomic, which carry the tagged *gTlr9* gene sequence. The pgTLR9A-Flag-genomic plasmid contains three consecutive Flag tags inserted before the stop codon in exon 3, and hence Flag-tagged gTLR9A protein will be generated from the mRNA that has intron 2 spliced out. The pgTLR9B-HA-genomic plasmid contains four consecutive HA tags inserted into the 5′ end of intron 2; hence, if intron 2 is not spliced, the translation process will continue into the intron and terminate at the stop codon within the intron to generate a translational product of gTLR9B tagged with HA. The plasmids were transfected into HEK293T cells to verify protein expression. As shown in Fig 2B, distinct bands at ~125 kDa and ~115 kDa, relating to tagged-gTLR9A and gTLR9B, respectively, were visualized by western blotting. These data demonstrate that the *gTlr9* gene can produce both *gTlr9A* and *gTlr9B* transcripts and the products of their translation through RNA splicing.

### Tissue distribution of *gTlr9A* and *gTlr9B*

We subsequently assessed whether the expression of the two *gTLR9* isoforms is a tissue-specific event. As shown in Fig 2C, both *gTlr9A* and *gTlr9B* were constitutively expressed in various tissues of healthy fish. The expression of *gTlr9A* and *gTlr9B* was predominantly observed in immune-associated tissues, such as the spleen, head kidney, as well as the liver. Of note, the level of *gTlr9A* was significantly higher than that of *gTlr9B* in all tissues, but the relative ratio between the molecules varied (Fig 2C). These results indicate that the expression of *gTlr9A* and *gTlr9B* might be differentially regulated.

### Overexpression of gTLR9B suppresses downstream genes expression

**Fig 3:**
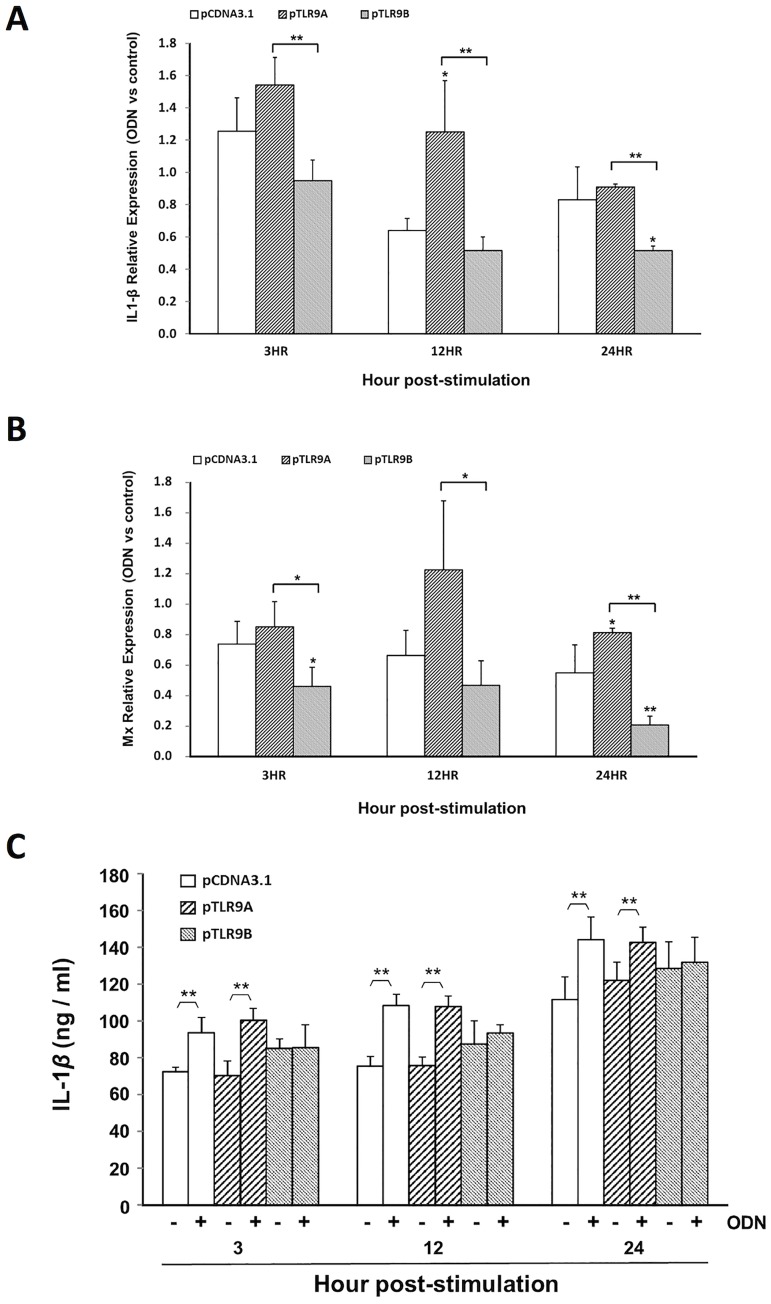
The inhibitory effect of gTLR9B on TLR9 signaling. GK cells were co-transfected with plasmids capable of expressing either gTLR9A (pcDNA3.1-9A) or gTLR9B (pcDNA3.1-9B) along with plasmid pRL-TK encoding *Renilla* luciferase as a transfection control using Lipofectamine 2000 (Invitrogen). *A*, Total RNA was harvested to measure the expression of genes downstream of TLR9 signaling. *B*, An ELISA assay was performed to determine IL-1β protein expression. All values were normalized to *Renilla* mRNA expression. *0.01< *p* < 0.05, **p < 0.01, Student’s *t*-test.

TLR isoforms can function as either positive or negative regulators of TLR signaling [30–32]. While it acts as a membrane protein with an intact N-terminal LRR-domain, gTLR9B has a deficient C-terminal TIR domain, which is important for the initiation of downstream signaling. We therefore hypothesized that gTLR9B might serve as a negative regulator of grouper TLR9 signaling. To test this hypothesis, we transiently transfected plasmids expressing either gTLR9A (pcDNA3.1-9A) or gTLR9B (pcDNA3.1-9B) cDNA. A control plasmid, pRL-TK, expressing *Renilla* luciferase was also included to normalize for transfection efficiency. As shown in Fig 3A and 3B, upon stimulation with the TLR9 agonist CpG ODN, *Il-1*β and *Mx* mRNA expression were upregulated at all time points in GK cells transfected with the control plasmid or the gTLR9A encoding plasmid. However, in gTLR9B-transfected cells, no increase in *Il-1*β and *Mx* mRNA expression was observed. The inhibition of *Il-1*β was also verified at the protein level. As shown in Fig 3C, overexpression of gTLR9A increased the quantity of IL-1β protein, whereas overexpression of TLR9B did not. The *Il-1*β mRNA level over time did not appear to match with the protein level. We reason that the discrepancy may be due to the shorter half-life of the *Il-1*β mRNA. Of note, our assay system only allows us to measure the steady-state expression of the *Il-1*β mRNA but the accumulated amount of the IL-1β protein. Overall, these results support the role of gTLR9B as a negative regulator of the TLR9 signaling pathway in grouper cells.

### Co-localization of gTLR9, adaptor protein, and ligand in the endosome after activation

Based on the structural features of gTLR9B, that is, an intact ligand-binding LRR domain but no signal-transducing TIR domain, the negative regulatory function of gTLR9B may arise due to its ability to act as a molecular sink by competing with gTLR9A for ligands. As proper compartmentalization of TLR9 in the endosome is essential for its ability to detect unmethylated CpG DNA, and for it to recruit the adaptor protein MyD88 (6), we investigated whether both gTLR9A and gTLR9B can translocate from the ER to the endosome upon stimulation. We therefore cloned grouper *MyD88* (*gMyD88*) cDNA, which is 1638 nt long and encodes a protein comprised of 289 amino acids that contains the signature death and C-terminal TIR domains. The nucleic acid sequence of gMyD88 shares 99.2% identity with a grouper MyD88 sequence deposited in the NCBI database (ID GQ202584).

**Fig 4:**
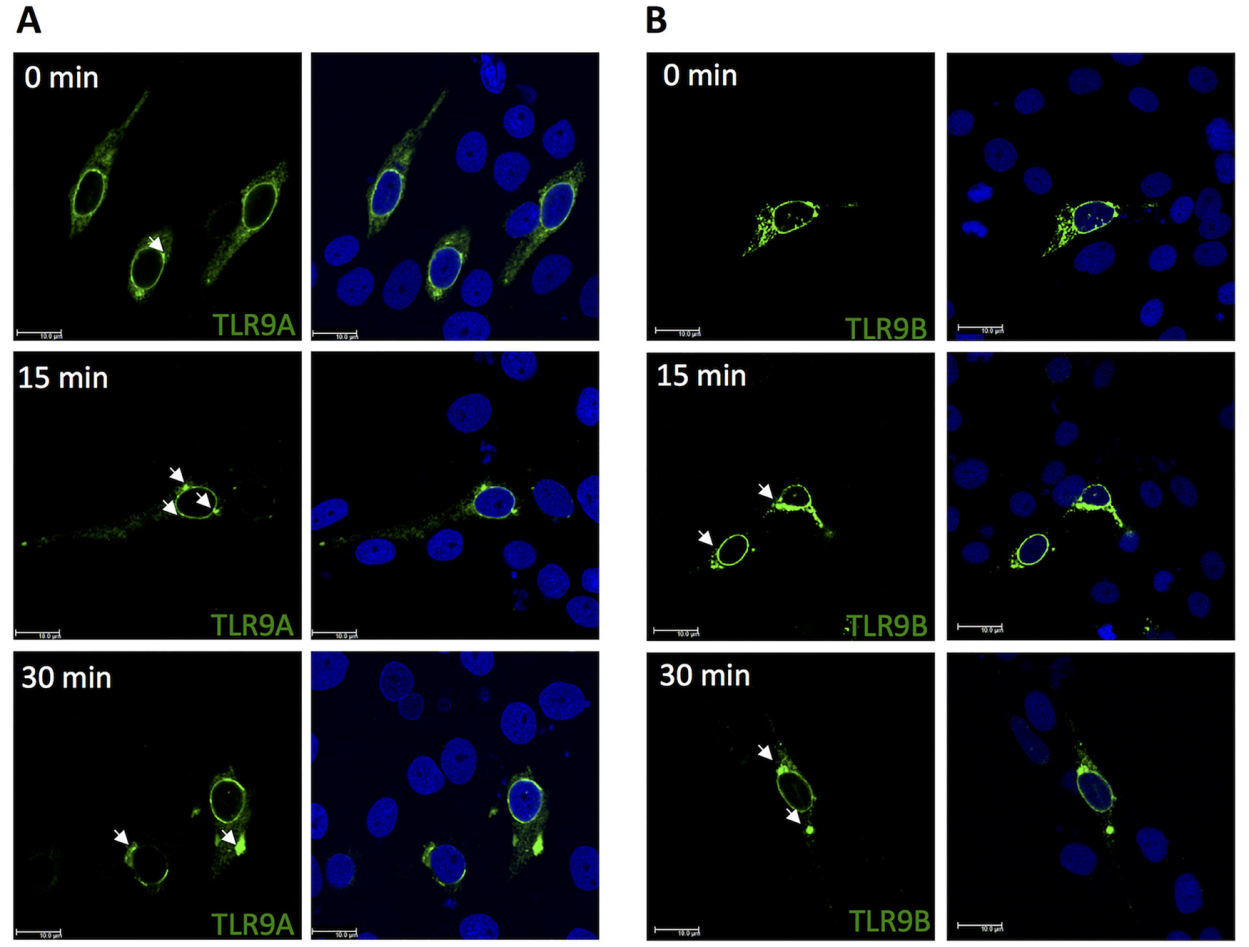
Mobilization of gTLR9A and gTLR9B upon stimulation with CpG ODN. (A) In non-stimulated cells (0 min), gTLR9 is primarily localized perinuclearly in a diffused interconnected web pattern resembling that of the ER. Upon stimulation with ODN, TLR9A gradually formed large aggregates at the location of the presumed endosome. (B) gTLR9B exhibited a pattern similar to that of gTLR9A. These results indicate that both gTLR9A and 9B can be mobilized from the perinuclear area to the endosome. The exact location of the endosome was verified as shown in Fig 6. Results are presented as the observation of at least 100 transfected cells from one representative experiment out of two.

Trafficking of gTLR9A and gTLR9B after stimulation with CpG DNA was assayed in GK cells transfected with plasmids expressing gTLR9A-Flag or gTLR9B-HA. The transfected cells were treated with CpG ODN. As shown in Fig 4, unstimulated Flag-tagged gTLR9A and HA-tagged gTLR9B showed a diffuse intracellular interconnected web pattern, which is similar to that of the ER, with occasional patches around the nucleus (indicated by a white arrow). At 15-min post-stimulation, the diffuse pattern of gTLR9A and gTLR9B localization was replaced by the formation of large speckles in the perinuclear region. At 30-min post-stimulation, the gTLR9A and gTLR9B speckles moved further away from the perinuclear region and formed larger aggregates at a location that was presumed to be the endosome. This observation indicates that gTLR9A and gTLR9B can respond to CpG ODN stimulation and translocate from the perinuclear ER region to the endosome.

**Fig 5:**
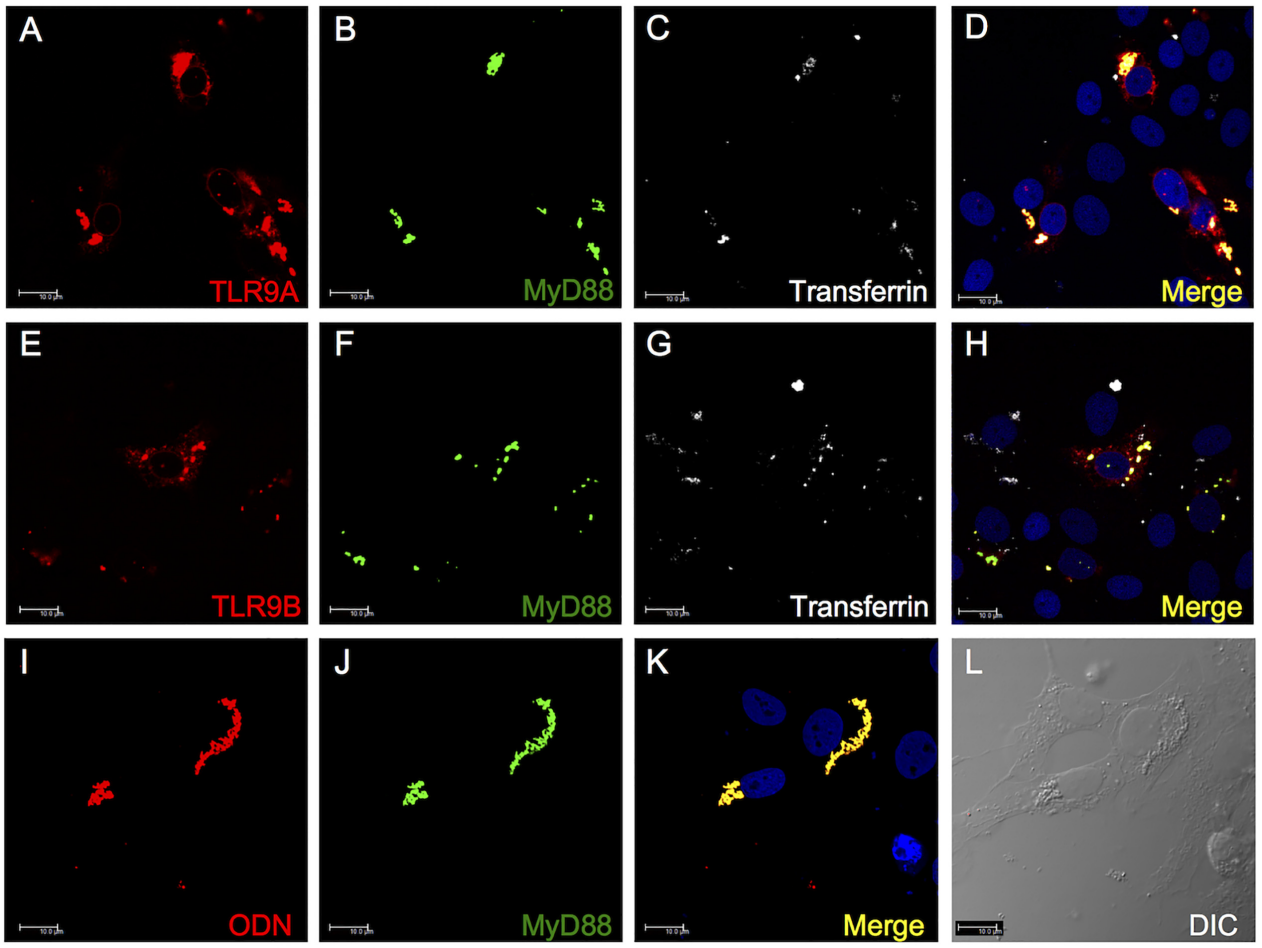
Sub-cellular co-localization of gTLR9A, gTLR9B, and gMyD88 in the endosome. GK cells were co-transfected with gTLR9A-Flag and YFP-gMyD88 (*A*–*D*) or gTLR9B-HA with YFP-gMyD88 (*E*–*H*). Transfected cells were incubated with the endosome marker transferrin conjugated to Alexa Fluor 633 along with CpG ODN for 30 min. Images were then acquired on an inverted confocal microscope. The merged images (*D* & *H*) demonstrate that gTLR9A and gTLR9B can translocate to the endosome and co-localize with gMyD88 upon stimulation with CpG ODN. The sub-cellular co-localization of CpG ODN and the receptor-adaptor complex was assayed in GK cells transfected with EYFP-MyD88 and treated with Texas-red-conjugated ODN. Both ODN (*I*) and gMyD88 (*J*) formed granular aggregates and co-localized with each other (*K*). The fluorescent granules exhibited structural features of organelles under differential interference contrast (DIC) microscopy (*L*). Scale bar: 10 μm. Results present the observation of 100 transfected cells or more from one representative experiment out of two.

The exact subcellular localization of gTLR9A and gTLR9B was further verified in GK cells transfected with plasmids expressing TLR9A-Flag, TLR9B-HA, and EYFP-MyD88. Twenty-four-hours after transfection, cells were stimulated with CpG ODN for 30 min. Similar to a previous observation, both gTLR9A and gTLR9B formed a diffuse pattern in the perinuclear region and large aggregates presumably at the location of the endosome (Fig 5A & 5E). EYFP-tagged gMyD88 was observed in small round aggregates (Fig 5B & 5F), and co-localized exclusively with gTLR9A and gTLR9B (Fig 5D & 5H). To confirm that the gMyD88- and gTLR9-enriched intracellular compartments were endosomes, transferrin conjugated to Alexa Fluor 633, a marker of early endosomes, was applied to GK cells following treatment with ODN. We found that gTLR9A, gTLR9B, gMyD88, and transferrin co-localized in the same speckle-like structure (Fig 5D & 5H), demonstrating that both gTLR9A and gTLR9B can be transported into the endosome, where they then co-localize with gMyD88.

### Interaction between gTLR9 receptors, adaptor protein, and ligand

**Fig 6:**
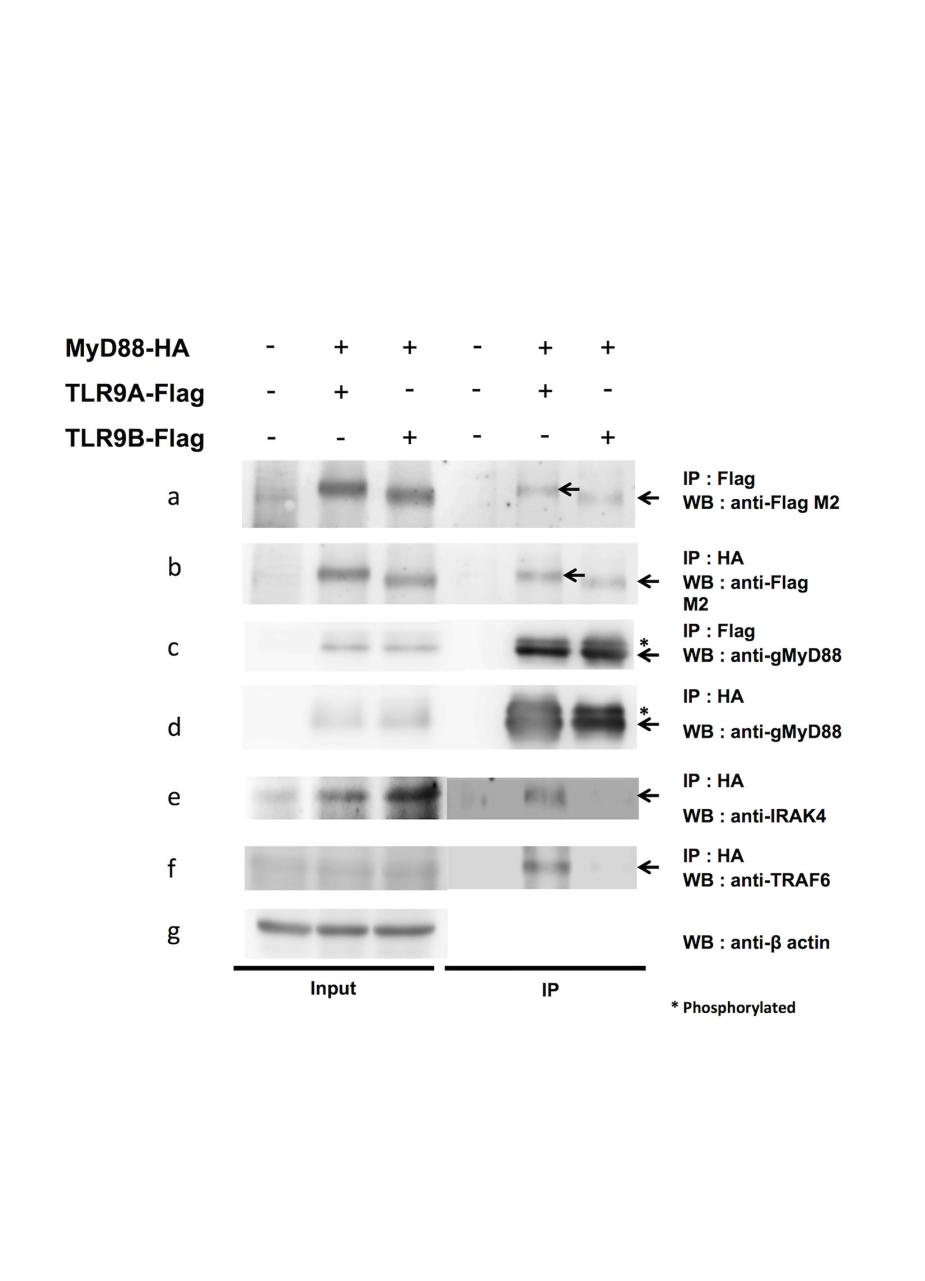
Assembly of gTLR9 signaling complexes. Molecular interactions between gMyD88, gTLR9A/9B, and downstream proteins were investigated by co-immunoprecipitation assay. HEK293T cells were co-transfected with gTLR9A/9B-Flag and gMyD88-HA. Immunoprecipitation was performed with cell lysates incubated with Dynabeads Protein G coated with anti-FLAG or anti-HA monoclonal antibodies. Precipitated proteins were detected using antibodies against Flag, HA, anti-IRAK4, and anti-TRAF6 antibodies. Left panel, total input protein; right panel, immunoprecipitated proteins. Antibodies used for immunoprecipitation and western blotting are listed on the right.

To verify that the ODN ligand also co-localized with the receptor-adaptor complex, EYFP-MyD88-transfected GK cells were treated with Texas-red-conjugated ODN. As shown in Fig 5I–5K, both gMyD88 and ODN formed granular aggregates and co-localized. The fluorescent granules were also found to exhibit structural features of organelles under differential interference contrast (DIC) microscopy (Fig 5L). To confirm there was indeed physical contact between ligand, receptor, and adaptor, a Co-IP assay was carried out with biotin-tagged CpG ODN and HEK293T cells co-transfected with a combination of gTLR9A- or 9B-Flag and gMyD88-HA. The results of the Co-IP showed that both gTLR9A and gTLR9B could bind to gMyD88, but the ability to interact with the downstream effectors, IRAK4 and TRAF6, was only identified for gTLR9A, and not gTLR9B (Fig 6, panels a, c and e, f). We also found that both gTLR9A and gTLR9B could interact with the TLR9 ligand CpG ODN, but not with the TLR3 ligand poly I:C (data not shown). Taken together, these results support our hypothesis that gTLR9B can compete with CpG ODN with gTLR9A in the endosome. These results also suggest that gTLR9 signaling can be inhibited by gTLR9B the inability of gTLR9B to recruit downstream IRAK4 and TRAF6.

### Orchestrated expression of *gTlr9A* and *gTlr9B* upon stimulation with TLR9 agonists

**Fig 7:**
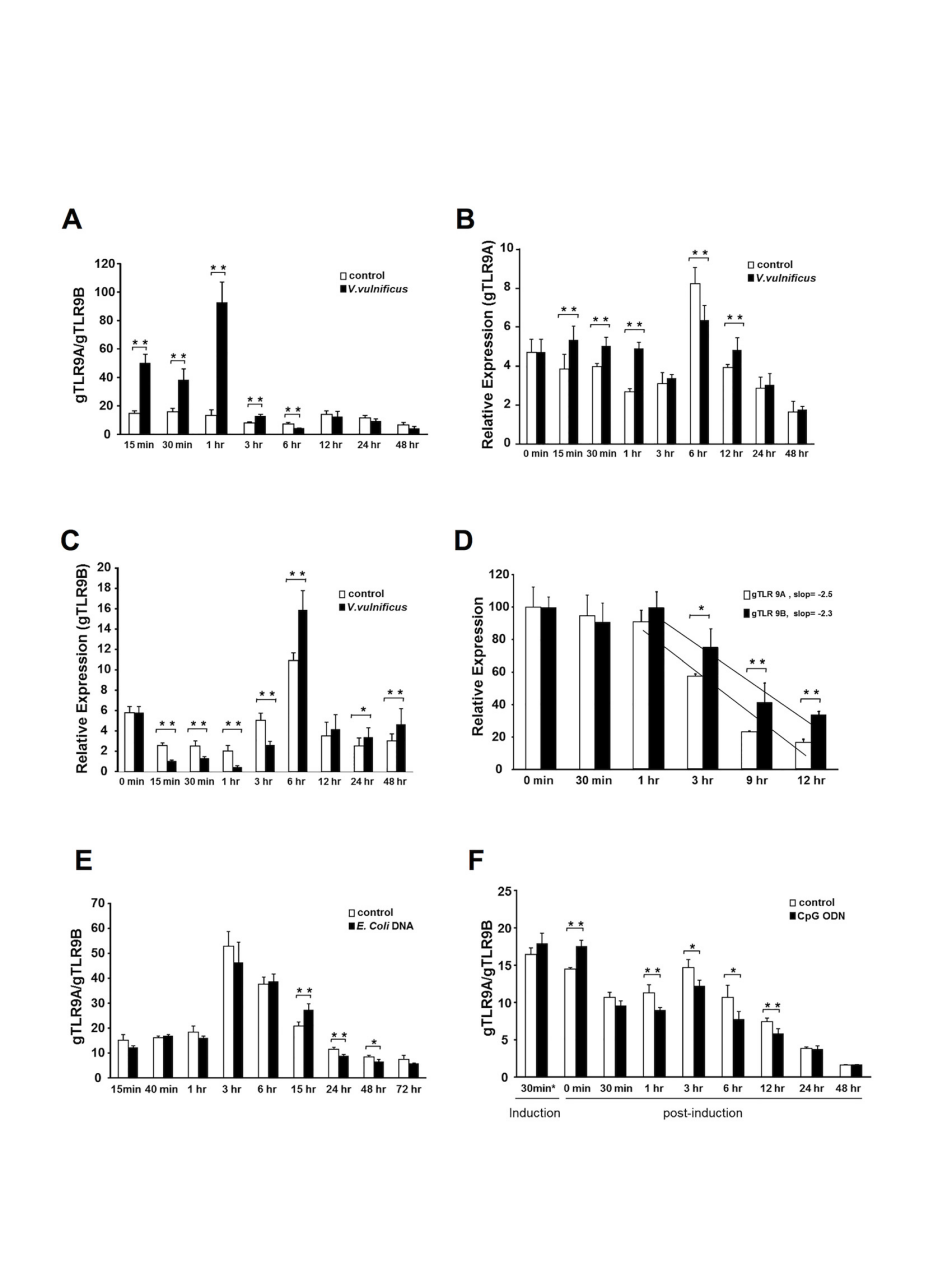
Differential Expression of *gTlr9A* and *gTlr9B* in GK cells upon immuno-stimulation. A–C, GK cells were stimulation with inactivated *V vulnificus*. The quantity of *gTlr9A* and *gTlr9B* mRNA in the treated and control cells was measured by RT-qPCR. *A*, After stimulation, the ratio of *gTlr9A/gTlr9B* increased during the first 3 h. *B*, *C*, Further analysis of *gTlr9A* and *gTlr9B* expression showed that the steady-state level of *gTlr9A* mRNA in GK cells promptly increased whereas that of *gTlr9B* was suppressed during the first 3 h; this pattern then reversed. *D*, To measure the stability of *gTlr9A* and *gTlr9B* mRNAs, 4-h after stimulation with *V*. *vulnificus*, cells were treated with actinomycin D to inhibit *de novo* transcription. The quantity of both *gTlr9* isoforms declined after 3 h at approximately the same rate. *E*, *F*, The relative expression of *gTlr9A* and *gTlr9B* mRNAs was determined in response to bacterial chromosomal DNA (*E*) and CpG ODN (*F*), two known TLR9 agonists. A similar pattern in the relative expression of the two isoforms was observed. Results are presented as the mean ± SD of triplicate samples. *0.01< *p* < 0.05, **p < 0.01, Student’s *t*-test.

Our *in-vitro* data showed that *gTlr9A* and *gTlr9B* transcripts could be generated via alternative splicing of pre-mRNA. Together with the inhibitory role of gTLR9B on downstream gene expression, we wished to determine whether the splicing event is regulated by the activation of gTLR9. To verify that *gTlr9A/9B* expression is differentially regulated, we measured the relative expression of the two isoforms in GK cells stimulated with formalin-inactivated *V*. *vulnificus*, which we previously found to be capable of inducing *gTlr9* expression in grouper cells (data not shown). As shown in Fig 7A–7C, after stimulation with inactivated bacteria, the steady-state level of *gTlr9A* mRNA expression in GK cells promptly increased, whereas that of *gTlr9B* was suppressed to a level lower than that observed in the non-stimulated cells. Over time, the level of *gTlr9B* mRNA increased, and was accompanied by a decrease in the level of *gTlr9A*. These data demonstrate the expression of *gTlr9A* and *gTlr9B* is differentially regulated upon immune stimulation

To investigate the possibility that the differential expression of *gTlr9A* and *gTlr9B* mRNA was not related to their stability, we examined temporal changes in the steady-state level of both mRNAs after stimulation. GK cells were stimulated with formalin-inactivated *V*. *vulnificus*, which was followed by the addition of actinomycin D to inhibit further *de-novo* transcription. The relative expression of *gTlr9A* and *gTlr9B* mRNA was then measured by RT-qPCR. As shown in Fig 7D, the quantity of both isoforms remained stable up to 1 h after the addition of actinomycin D and started to decline after 3 h at approximately the same rate. Therefore, the differential expression of the two RNA isoforms was unlikely to be due to differences in their stability.

We further investigated the expression profile of *gTlr9A* and *gTlr9B* mRNAs in response to two other TLR9 agonists: bacterial chromosomal DNA and CpG ODN. A similar trend in the *gTlr9A/gTlr9B* ratio was observed (Fig 7E and 7F). Taken together, these data support a scenario where the expression of *gTlr9A* and *gTlr9B* mRNAs is strictly regulated via alternative splicing of RNA in response to immunostimulation. Consequently, this mechanism may play a critical role in the regulation of gTLR9 signaling because alternatively spliced gTLR9B can serve as a negative regulator of the pathway.

## Discussion

Post-transcriptional regulation, such as pre-mRNA AS, is gradually being recognized as a mechanism through which organisms can increase the complexity of immune regulation. AS regulates gene expression by generating multiple distinct mRNAs from a given gene via a process in which exon(s) or intron(s) are skipped or introduced, leading to the instability of mRNA or the production of proteins with altered function [33]. Here, we show that grouper TLR9 exists in two isoforms, gTLR9A and gTLR9B. The gTLR9A isoform contains all of the essential elements that are characteristic of TLRs, including an N-terminal LRR domain, a transmembrane domain, and a C-terminal TIR domain. The gTLR9B isoform is identical to gTLR9A but lacks the αD, βE, αE, and parts of the connective DD loop in the TIR domain. In fact, the genomic structure of *TLR9*, unlike that of *TLR3*, is less conserved than that of the other TLRs across vertebrates, as characterized by the numbers of introns, which range from zero to four (Fig 1A). In fish examined thus far that exhibit a two-intron *Tlr9* gene structure all undergo intron 2-retention AS, as indicated by the presence of the same two TLR9 isoforms. Yet, there is no evidence to indicate that TLR9B transcripts undergo nuclear exportation and translation into protein products. Furthermore, there is no evidence to suggest what the role of TLR9B might be in the regulation of TLR9 signaling. We sought to address these issues by first analyzing the transcriptional and translational capacity of the *gTlr9* gene. These results demonstrate that both *gTlr9A* and *gTlr9B* mRNA and protein can be generated from the *gTlr9* gene via RNA AS.

Unlike the liver-specific expression of the soluble TLR5 isoform in some teleosts [29], both grouper *Tlr9* mRNA isoforms were identified predominantly in the head kidney, liver, and spleen (Fig 2C), a pattern that reflects the importance of fish TLR9 in immunity, similar to that of its human counterpart [34]. Noticeably, we have shown that the ratio of g*Tlr9A*/g*Tlr9B* expression varied in grouper tissues. However, this result is not consistent with the previously characterized *Sparus aurata* and *Psetta crocea Tlr9A* and *Tlr9B* [21,24], in which no variation was detected in the relative expression of each isoform. We suggest that this discrepancy might be due to differences in the species or assay method.

The lack of an intact signal transducing TIR domain in gTLR9B led us to hypothesize that gTLR9B could negatively regulate gTLR9 signaling. Nonetheless, not all of the truncated TLR isoforms identified so far serve as negative regulators, for example salmonid TLR5 [29]. However, we observed a decrease in the level of downstream *Il-1*β and *Mx* mRNA expression and IL-1β protein expression in gTLR9B-transfected cells as compared with cells transfected with gTLR9A or an empty vector (Fig 4). Furthermore, we observed that gTLR9A and gTLR9B translocated to the early endosome in response to ODN stimulation (Fig 4), along with the downstream adaptor MyD88 (Fig 5D and 5H). Together, these data support the hypothesis that both gTLR9A and gTLR9B participate in TLR9 signaling and that gTLR9B serves as a negative regulator of this signaling pathway.

Examples of negative regulatory roles of alternatively spliced TLR variants have been reported in mammalian systems. For example, alternatively spliced human TLR3 lacks 64 amino acids between LRR10 and LRR12. This splice variant has been shown to be defective in TLR3 signaling following stimulation with poly I:C in 293T cells overexpressing the alternatively spliced TLR3. [32,35]. Another example is the alternative splice variant of mouse TLR4, which encodes a 20-kDa soluble protein containing only the first 86 a.a of the extracellular LRR domain. Soluble TLR4 can inhibit the LPS-mediated increase in NF-κB and TNF-α production in mouse macrophage RAW264.7 cells [31]. In human, TLR4 also undergoes alternative splicing and produce soluble TLR4 isoform. The soluble TLR4 isoform expression after exposure to LPS is significantly less in monocytes from patients with cystic fibrosis (CF). The monocytes from CF patients with lower soluble TLR4 display a higher level of TNF than do those from healthy patients. This example highlights a physiological role of TLR variants in limiting innate immune responses and creates a potential feedback regulatory loop in immune cells [36]. Unlike the alternative variants of mammalian TLR3 and TLR4, with which contain defects in the N′-terminal ecodomain, gTLR9B contains a structural defect located within the C′-terminal TIR domain, suggesting that its regulatory mechanism is involved in the interaction with downstream factors rather than the interaction with ligands. Several reports have demonstrated that a truncated TIR may abolish the function of the TLR. For instance, when the last 32 amino acids were deleted, human TLR9 could abolish CpG ODN-dependent cell activation [5]. Another example is RP105, a TLR4 homolog that shares all of the characteristics of TLRs except for the C-terminal TIR domain [37]. RP105 could suppress TLR4 signaling in HEK293T cells [37,38]. Interestingly, in response to LPS stimulation, the increase in RP105 expression was delayed when compared with that of *TLR4* in mouse and human dendritic cells as well as in macrophages. This delayed expression is similar to that observed for *gTlr9B*. Nonetheless, our study further revealed a temporal difference in expression of the two grouper *Tlr9* isoforms (Fig 7A–7C). More importantly, we have shown that the differential expression of *gTlr9A* and *gTlr9B* is not a result of selective degradation of one particular mRNA species over the other, as evidenced by the same rate of *gTlr9A* and *gTlr9B* mRNA degradation in the presence of actinomycin D (Fig 7D) Taken together, these results support a temporal nature of the regulatory role of gTLR9B in gTLR9 signaling.

The regulatory function of gTLR9B might arise from the competition for ligands against gTLR9A and/or the inability to interact with the downstream adaptor(s). Confocal imaging showed that both gTLR9A and gTLR9B could co-localize individually with CpG ODN in the endosome, suggesting that both gTLR9 isoforms can interact with this ligand. A co-immunoprecipitation assay was performed to examine the assembly of the TLR9 signaling complex. We observed that both gTLR9A and gTLR9B retained the ability to interact with gMyD88, while gTLR9B failed to recruit IRAK4 and TRAF6 (Fig 6). Agonist binding induces the dimerization of both TLR ectodomains and TIR domains [39]. Structurally, gTLR9B TIR lacks the αD, βE, αE and is defective in the DD loop (Fig 1B). Studies investigating the crystal structure of the human TLR10 TIR domain, have demonstrated that TLR TIR-TIR dimer formation mainly involves the BB loop, CC helix and DD loop [40,41]. On the other hand, information regarding the structure of the TLR2-MyD88 interaction reveals that TLR2 interacts with MyD88 through an interaction between the BB loop located on TLR TIR, and the DD loop located on MyD88 TIR. Therefore, we speculate that truncated gTLR9B might affect gTLR9 TIR-TIR dimerization but not the recruitment of MyD88. This hypothesis is consistent with our observation that both gTLR9A and gTLR9B could interact with gMyD88. MyD88 is an adaptor protein that contains an N-terminal death domain and a C-terminal TIR domain for homotypic interactions that bridge the upstream receptor and downstream signaling proteins. Once recruited, MyD88 leads to the formation of a large Myddosome complex, which contains kinases that are involved in downstream signaling. Structural analysis of human MyD88 indicates that at least four MyD88 units are required to form a platform for the downstream death domain-containing kinase IRAK4 to dock. In this regard, proper formation of the TLR dimer or even higher-order TLR multimers may be crucial for Myddosome assembly [42]. A previous study demonstrated that TLRs can be recruited to lipid rafts, which would favor higher-order receptor complex formation [43]. We propose that gTLR9B can interact with gMyD88, but may fail to form TLR TIR-TIR dimers due to the defective DD loop. Consequently, gTLR9B might fail to form a higher-order structure, leading to improper assembly of the Myddosome, which might, therefore, abolished IRAK4 docking. Taken together, we hypothesized that gTLR9B interferes with gTLR9 signaling by competing with ligand with gTLR9A, and impairing the gTLR9 multimer formation that is required for Myddosome recruitment of the downstream kinase IRAK4, and TRAF6. Although we provide a possible scenario to explain how gTLR9B interferes with gTLR9 signaling, we do not rule out the possibility that gTLR9B may affect conformational changes induced by gTLR9 dimerization in both gTLR9 and gMyD88. These changes in conformation may subsequently affect the formation of a signaling complex, thus preventing the binding of downstream proteins.

## Conclusion

Here, we propose a mechanism to explain how grouper TLR9 signaling is orchestrated by the alternatively spliced isoform gTLR9B. The mechanism encompasses rapid elevation of *gTlr9A* expression when ligands are encountered to initiate an immune response, while the production of *gTlr9B* is later favored by RNA AS to down-regulate signaling by functioning as a molecular sink to compete against gTLR9A for ligand association in the endosome. We also demonstrated that gTLR9B interferes with the assembly of a functional signaling platform. Despite the findings presented here, more questions are raised, such as the stoichiometry of gTLR9 signaling complex assembly (i.e., gTLR9A-gTLR9A or gTLR9A-gTLR9B dimer or higher-order structure formation), the competition between gTLR9A and gTLRB with downstream adaptor gMyD88, and how this regulation is coordinated with the molecular mechanism of RNA AS. Further studies are ongoing in our laboratory to address these questions. Finally, we speculate that this AS-mediated mechanism in gTLR9 signaling might also occur in other teleosts that harbor TLR9 with the same genomic organization. To date, 2-intron genomic organization has only been observed in members of the orders *Perciformes* and *Pleuronectiformes* (Fig 1A & 1C), two of the most evolutionally advanced fish orders, indicating that this regulatory mechanism was acquired relatively recently in the evolution of fish.

## Supporting Information

S1 Fig Sequence of grouper *Tlr9*. The full-length gTLR9 cDNA is 3800 nucleotides (nt) in length, consisting of a 265-nt 5ʹ-untranslated region (UTR), a 317-nt 3′-UTR and an open reading frame encoding a protein of 1061 amino acids. Prediction of protein domains revealed a potential signal peptide (thin dash line) at positions 1–20 of gTLR9, a putative transmembrane domain (thick dash line) between residues 842 and 864, 14 leucine-rich repeats (LRRs, thick line), and an intracellular C-terminal region showing a typical TIR domain (triple line). The CXXC-containing motifs that are important for direct binding to the unmethylated CpG ODNs, are marked by a double line. Three conserved boxes (box I, II, and III), which are important for TLR function, are highlighted. The amino acid sequence is shown in capital letters.
(PDF)

S2 Fig Verification of grouper IL-1β antibody specificity. IL-1β recombinant protein (0–100 ng) was resolved by SDS-PAGE and then transferred to PVDF membrane for western blotting. A rabbit-anti-grouper IL-1β antibody made in-house and diluted in 0.5% PBS-T containing 5% milk was used as the primary antibody.
(PDF)

S1 Table Primers used in this study. (PDF)

S2 Table Comparison of identities and similarities of grouper TLR9 isoform A and its counterparts in other species. (PDF)
