# Supplementary material for: Toll-Like Receptor 9 Alternatively Spliced Isoform Negatively Regulates TLR9 Signaling in Teleost Fish
Source: PLoS One. 2015 May 8;10(5):e0126388. doi: 10.1371/journal.pone.0126388 (PMC4425437; doi:10.1371/journal.pone.0126388)
Supplement: S1 Fig — The full-length gTLR9 cDNA is 3800 nucleotides (nt) in length, consisting of a 265-nt 5ʹ-untranslated region (UTR), a 317-nt 3′-UTR and an open reading frame encoding a protein of 1061 amino acids. Prediction of protein domains revealed a potential signal peptide (thin dash line) at positions 1–20 of gTLR9, a putative transmembrane domain (thick dash line) between residues 842 and 864, 14 leucine-rich repeats (LRRs, thick line), and an intracellular C-terminal region showing a typical TIR domain (triple line). The CXXC-containing motifs that are important for direct binding to the unmethylated CpG ODNs, are marked by a double line. Three conserved boxes (box I, II, and III), which are important for TLR function, are highlighted. The amino acid sequence is shown in capital letters. (PDF) [file pone.0126388.s001.pdf]

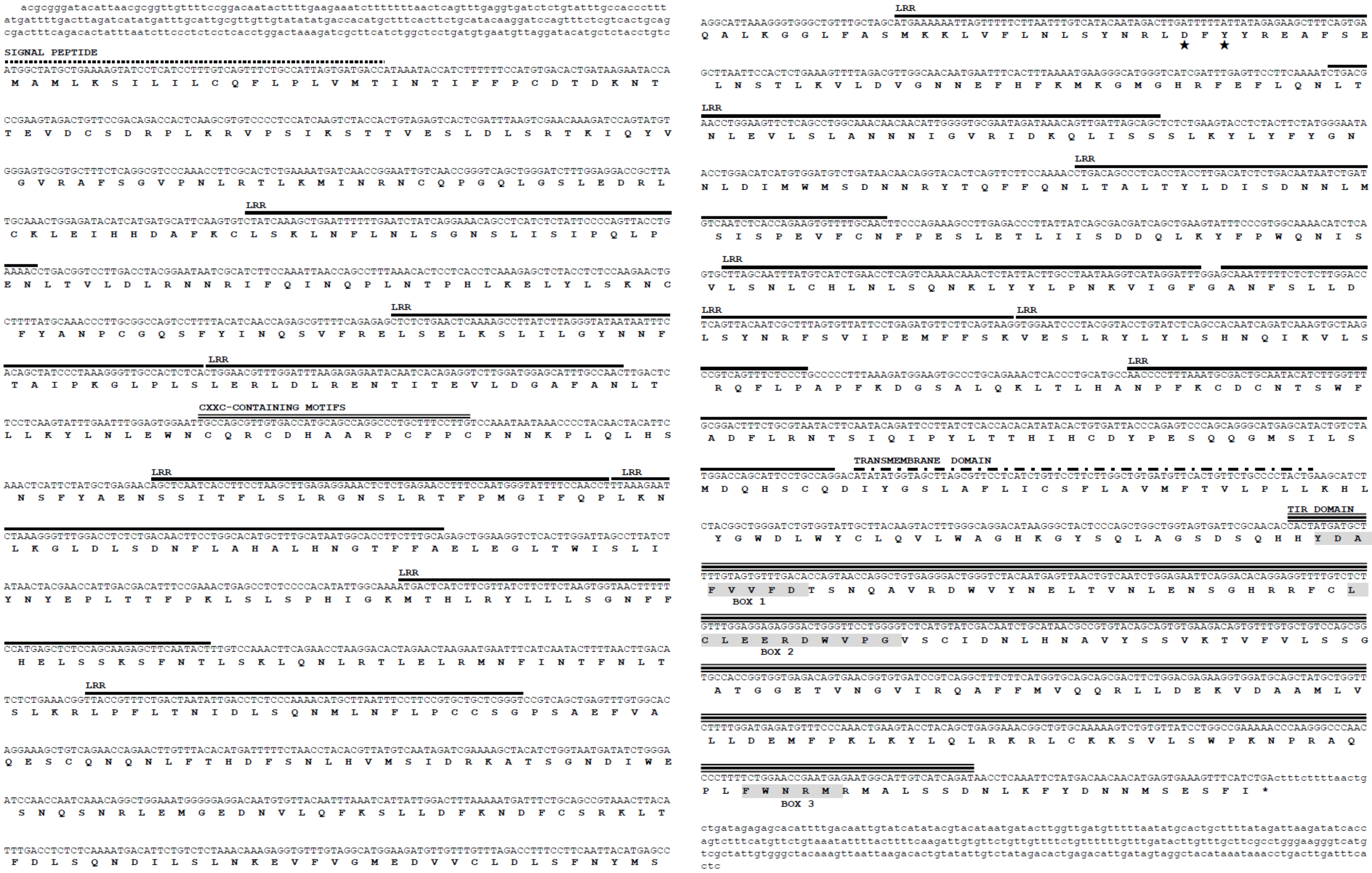
SIGNAL PEPTIDE
LRR
CXXC-CONTAINING MOTIFS
TRANSMEMBRANE DOMAIN
TIR DOMAIN
BOX 1
BOX 2
BOX 3

S1.Fig.
