# Supplementary figures and images for: Toll-Like Receptor 9 Alternatively Spliced Isoform Negatively Regulates TLR9 Signaling in Teleost Fish
Source: PLoS One. 2015 May 8;10(5):e0126388. doi: 10.1371/journal.pone.0126388 (PMC4425437; doi:10.1371/journal.pone.0126388)

Recombinant  
grouper IL-1 $\beta$ +lysate

---

0 ng

25 ng

50 ng

100 ng

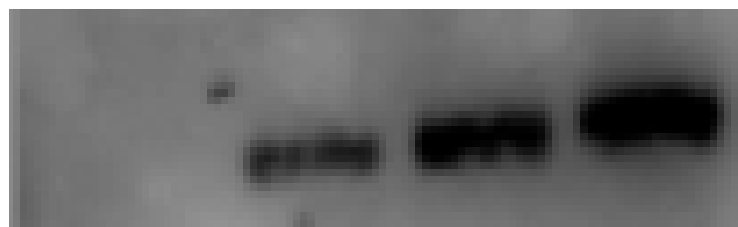

S2.Fig.

Supplement: S2 Fig — IL-1β recombinant protein (0–100 ng) was resolved by SDS-PAGE and then transferred to PVDF membrane for western blotting. A rabbit-anti-grouper IL-1β antibody made in-house and diluted in 0.5% PBS-T containing 5% milk was used as the primary antibody. (PDF) [file pone.0126388.s002.pdf]
