# Supplementary material for: Toll-Like Receptor 9 Alternatively Spliced Isoform Negatively Regulates TLR9 Signaling in Teleost Fish
Source: PLoS One. 2015 May 8;10(5):e0126388. doi: 10.1371/journal.pone.0126388 (PMC4425437; doi:10.1371/journal.pone.0126388)
Supplement: S1 Table — (PDF) [file pone.0126388.s003.pdf]

S1 Table

(A) TLR9

Set	Forward(F)	Reverse(R)	Note
TLR9-a (PC0040+PC0041)	5’CTCTACGGCTGGGAYYT3’	5’GTGGWCAYCACAAACACTG3’	D/D
TLR9-b (PC0191+PC0093)	5’AGCCTCAYSTACCTGGACAT	5’GTGTTGCGAATCACTACCAGCCA3’	D/S
TLR9-c (PC0134+PC0193)	5’TTCAGGACACAGGAGGTTTTGTC3’	5’CTYTCACTCATGTTGTTGTC3’	S/D
TLR9-d (PC0189+PC0192)	5’TGACCTSTCTCAAAATGACAT3’	5’ATTGTRRCTGAGGTCCAAGAG3’	D/S
TLR9-e (PC0216+PC0217)	5’ATCTRTCAGGAAAYAGYCTCA3’	5’AGGARGTTGTCMGAGAGGTCCA3’	D/D
TLR9-f (PC0228+PC0229)	5’GGAAACTCTCTGAGAACCTTTCCAAT3’	5’CTAAACAAACAACATCTTCCATGCCTAC3’	S/S
TLR9-g (PC0207+3’race)	5’ACCGAATGAGAATGGCATTGTCATCAGA3’	5’GACTCGAGTCGACATCGA3’	S/3R
TLR9-h PC0227+5’race	A*	5’TAATTTGGAAGATGCGATTATTCCGTAG3’	5R/S
TLR9-i (PC0268+PC0269)	5’ATGTGAATGTTAGGATACATGCTCTACC3’	5’TCAGATGAAACTTTCACTCATGTTGTTG3’	S/S
TLR9- full PC0629+PC0630	5’ACGCGGGATACATTAACGCGGTTGTT3’	5’GAGTGAAATCAAGTCAGGTTTATTTATG3’	S/S
TLR9- q (PC0344+PC0345)	5’CGTGTCCCCTCCATCAAGTCTA3’	5’ACTCCCACATACTGGATCTTTGTTC3’	S/S(ǂ)
TLR9-A- ORF PC0760+PC0761	5’ATGGCTATGCTGAAAAGTATCCTCATCC3’	5’TCAGATGAAACTTTCACTCATGTTGTTG3’	S/S
TLR9-A form-q PC0710+PC0711	5’GTTTGTGCTGTCCAGCGGT3’	5’GCATAGCTGCATCCACCTTCTC3’	S/S(ǂ)
TLR-B ORF PC0760+PC0762	5’ATGGCTATGCTGAAAAGTATCCTCATCC3’	5’TCACACCCACAAAATACATGTGAGAATA3’	S/S
TLR9-B form-q PC0691+PC0692	5’GCGACTTCTGGACGAGAAGGT3’	5’AACATGGCTACAACAGGATATGAATC3’	S/S(ǂ)
TLR9A,B form PC0683+PC0684	5’GAAGACAGTGTTTGTGCTGTCCAGCGGT3’	5’GAGGTTATCTGATGACAATGCCATTCTC3’	S/S

(B) MyD88

Set	Forward(F)	Reverse(R)	Note
MyD88-a PC0078+PC0079	5’TTGATGCYTTCATCTGCTACTG3’	5’TGGCCAGYCGDRYCCAGAACCA3’	D/D
MyD88-b 5’-Race+PC0200	A*	5’ACAGCTTCAGCCTGCACTCCGTCTGCTC3’	5R/S
MyD88-c PC0111+3’-Race	5’ACCAAGCCTTTGACACAGAG3’	5’GACTCGAGTCGACATCGA3’	S/3R
MyD88-ORF PC0212+PC0213	5’ATGGCGTGTAAGGACCCAGA3’	5’TTACGGCAGCGAGAGCGCCT3’	S/S
MyD88- full PC0631+PC0632	5’ACGCGGGGGAAGTGTGGGACAACTCAGT3’	5’AAGTGTGTGAAAATTTATTTTGAGAA3’	S/S
MyD88-q PC0264+PC0265	5’GCGACGCCTGTGACTTTCA3’	5’TTCGAGCTCCGGGACAGA3’	S/S(ǂ)

(C) Other genes

Set	Forward(F)	Reverse(R)	Note
β-actin PC0247+PC0248	5’CCTGACAGAGCGTGGCTACTC3’	5’CCTTGATGTCACGCACGATT3’	S/S
IL-1β PC0274+PC0275	5’GTGTTCGGTTGTGAGGCAACT3’	5’ GCGCGTCCTGGTGATCA3’	S/S
MX PC0253+PC-254	5’ CATTGCCAGGGTGGCTGTA3’	5’TGAACCTCTGGATCAGTCCCTTT3’	S/S

A*: 5' -CTAATACGACTCACTATAGGGCAAGCAGTGGTATCAACGCAGAGT- 3' (SMARTer^{TM} RACE cDNA Amplification Kit, Clontech), D: degenerate primer, 3R: 3’-RACE primer, 5R: 5’RACE-primer, S: gene-specific primer, S(ǂ): gene-specific qPCR primer
