# Supplementary material for: Toll-Like Receptor 9 Alternatively Spliced Isoform Negatively Regulates TLR9 Signaling in Teleost Fish
Source: PLoS One. 2015 May 8;10(5):e0126388. doi: 10.1371/journal.pone.0126388 (PMC4425437; doi:10.1371/journal.pone.0126388)
Supplement: S2 Table — (PDF) [file pone.0126388.s004.pdf]

S2 Table

Species	Nucleotide	Amino acid		GenBank ID
	Indentity (%)	Indentity (%)	Similarity(%)	
Epinephelus coioides isoform A	100	100	100	GQ358201
Larimichthys crocea isoform A	75	74	80	EU655704
Paralichthys olivaceus	71	69	76	AB234024
Dentex tumifrons	70	75	80	EU256335
Pagrus major	69	74	80	EU256334
Sparus aurata isoform A	69	74	80	AY751797
Acanthopagrus schlegelii	68	74	80	EU256333
Takifugu rubripes	61	66	75	AC156439
Oncorhynchus mykiss	59	60	70	NM_001129991
Salmo salar	56	60	70	NM_001123653
Danio rerio	51	51	63	NM_001130594
Canis lupus familiaris	45	34	46	NM_001002998
Felis catus	44	33	45	NM_001009285
Mus musculus	47	33	46	NM_031178
Homo sapiens	42	34	47	NM_017442
